# Signaling Properties of Chemerin Receptors CMKLR1, GPR1 and CCRL2

**DOI:** 10.1371/journal.pone.0164179

**Published:** 2016-10-07

**Authors:** Olivier De Henau, Gaetan-Nagim Degroot, Virginie Imbault, Virginie Robert, Cédric De Poorter, Saria Mcheik, Céline Galés, Marc Parmentier, Jean-Yves Springael

**Affiliations:** 1 Institut de Recherche Interdisciplinaire en Biologie Humaine et Moléculaire (IRIBHM), Université Libre de Bruxelles (ULB), Brussels, Belgium; 2 Institut des Maladies Métaboliques et Cardiovasculaires, Institut National de la Santé et de la Recherche Médicale, Université Toulouse III Paul Sabatier, Toulouse, France; 3 Walloon excellence in life sciences and biotechnology (Welbio), Wavre, Belgium; Indian Institute of Technology Kanpur, INDIA

## Abstract

Chemerin is a small chemotactic protein originally identified as the natural ligand of CMKLR1. More recently, two other receptors, GPR1 and CCRL2, have been reported to bind chemerin but their functional relevance remains poorly understood. In this study, we compared the binding and signaling properties of the three human chemerin receptors and showed differences in mode of chemerin binding and receptor signaling. Chemerin binds to all three receptors with low nanomolar affinities. However, the contribution of the chemerin C-terminus to binding efficiency varies greatly amongst receptors. By using BRET-based biosensors monitoring the activation of various G proteins, we showed that binding of chemerin and the chemerin 9 nonapeptide (^149^YFPGQFAFS^157^) to CMKLR1 activates the three G_αi_ subtypes (G_αi1_, G_αi2_ and G_αi3_) and the two G_αo_ isoforms (G_αoa_ and G_αob_) with potencies correlated to binding affinities. In contrast, no significant activation of G proteins was detected upon binding of chemerin to GPR1 or CCRL2. Binding of chemerin and the chemerin 9 peptide also induced the recruitment of β-arrestin1 and 2 to CMKLR1 and GPR1, though to various degree, but not to CCRL2. However, the propensity of chemerin 9 to activate β-arrestins relative to chemerin is higher when bound to GPR1. Finally, we showed that binding of chemerin to CMKLR1 and GPR1 promotes also the internalization of the two receptors and the phosphorylation of ERK1/2 MAP kinases, although with a different efficiency, and that phosphorylation of ERK1/2 requires both G_αi/o_ and β-arrestin2 activation but not β-arrestin1. Collectively, these data support a model in which each chemerin receptor displays selective signaling properties.

## Introduction

Chemerin is a small chemotactic protein originally identified by our laboratory and other groups as the natural ligand of CMKLR1/ChemR23 [1–3]. Chemerin is secreted as a 143-amino acid inactive precursor, pro-chemerin, and is activated by the proteolytic removal of six to seven amino acids from its C-terminus by proteases such as elastase or cathepsin G [4–6]. The nonapeptide chemerin 9 (^149^YFPGQFAFS^157^) derived from the C-terminus of the processed form of chemerin was also identified as a potent agonist of CMKLR1 [4]. CMKLR1 is coupled to the G_i/o_ family of G proteins and in cells expressing CMKLR1, chemerin inhibits cAMP production and promotes phospholipase C activation, IP_3_ release, calcium mobilization as well as activation of PI3K and MAPK pathways [7]. Chemerin also induces the recruitment of β-arrestin2 to CMKLR1 but it is not known whether this recruitment is independent from G protein activation. *Ex vivo*, chemerin promotes chemotaxis of leukocyte populations that express CMKLR1, including dendritic cells, macrophages and natural killer cells [1,8–10]. More recently, two other receptors, GPR1 and CCRL2, were reported to bind chemerin with high affinity, but very few data are available regarding the functional relevance of these two receptors [11–13]. GPR1 is the closest homolog of CMKLR1, sharing more than 40% sequence identity with CMKLR1. Expression of GPR1 was reported in the central nervous system, skin and adipose tissue as well as in few cell types including Leydig cells and granulosa cells [14–17]. GPR1 was identified as a chemerin receptor as a result of a screening involving the Tango assay that relies on arrestin recruitment [13]. Chemerin binding to GPR1 was also shown to activate weakly calcium mobilization in recombinant cell lines, but it is still unclear whether GPR1 triggers signaling cascades in native environments. Chemerin binding to GPR1 induces its rapid down-regulation, suggesting that it may be a scavenging receptor controlling extracellular chemerin levels [13]. CCRL2 was identified as a third chemerin receptor in experiments based on binding assays rather than functional output [11]. CCRL2 exhibits low sequence homology with CMKLR1 and GPR1 but is much more related to chemokine receptors, sharing more than 40% sequence identity with CCR1, CCR2, CCR3 and CCR5 [18]. CCRL2 is expressed by several leukocyte populations, including macrophages and dendritic cells, and its expression is increased following stimulation by liposaccharides, poly(I:C) or IFN-γ [19]. It has been claimed that CCRL2 promotes calcium mobilization and chemotaxis in response to chemokines CCL2, CCL5, CCL7 and CCL8 [20]. Later on, CCRL2 was also reported to bind CCL19 in the absence of signaling [21,22]. However, these results supporting a role of CCRL2 as a chemokine receptor were not confirmed by other groups [23]. Chemerin binding to CCRL2 does not induce calcium mobilization or ligand internalization [11]. The current model assumes that CCRL2 would be an atypical receptor devoid of signaling capacity, but able to increase the local concentration of chemerin and presenting the ligand to leukocytes expressing CMKLR1 [11,24]. Besides it role as a chemotactic protein that induces migration of leukocytes, chemerin was also reported as an adipokine and a growth factor [25]. The chemerin system is also associated to several pathologies including obesity, diabetes, psoriasis or tumorigenesis, but the role of chemerin and the contribution of each receptor to these pathologies remain to be determined precisely [26]. Therefore, identifying the properties of chemerin receptors is crucial to appreciate the role of the chemerin system *in vivo*. Chemerin receptors are expressed in different cell types and it is well known that the cellular context may introduce a bias for the comparison of their individual properties [27]. In the present study, we generate cell lines expressing each chemerin receptor and compare their binding and signaling properties independently of the cellular context. We show that although all three receptors bind chemerin with high affinity, the role of the chemerin C-terminus in the interaction is different for each receptor. By using BRET biosensors, we also show that chemerin and the chemerin 9 nonapeptide induce the activation of the three G_αi_ and the two G_αo_ subtypes as well as the recruitment of β-arrestin1 and 2. Finally, we demonstrate that binding of chemerin to CMKLR1 and GPR1 induces the phosphorylation of MAP kinases through non-exclusive G_i/o_ and β-arrestin2-dependent pathways.

## Materials and Methods

### Ethics statement

The experiments using animal samples were carried out in strict accordance with the national, European (EU Directives 86/609/EEC) and international guidelines in use at the Université Libre de Bruxelles and in accordance with the Helsinki Declaration. All procedures were reviewed and approved by the local ethic committee (Commission d’Ethique du Bien-Etre Animal, CEBEA) of the Université Libre de Bruxelles (Permit Number: 222N and 341N). All efforts were made to minimize suffering.

### Reagents, plasmids and cell lines

Chemerin and antibodies used for the detection of human CMKLR1 and CCRL2 receptors were purchased from R&D Systems. Home-made mouse anti-GPR1 antibodies were generated by DNA immunization and the specificity of the antibody confirmed by FACS analyses using CHO-K1 cells expressing control GPCRs (J.D. Fransen, Euroscreen). Chemerin 9 peptide (^149^YFPGQFAFS^157^) corresponding to the C-terminal end of the bioactive human chemerin was custom synthesized by Genescript. Plasmids encoding the G protein and arrestin 2 constructs were described previously [28]. Plasmids encoding β-arrestin1-Rluc and β-arrestin1-EYFP were kindly provided by S. Marullo [29]. MEF cell lines derived from β-arrestin knockout mice were provided by R. Lefkowitz [30]. CHO-K1 cells were cultured in Ham’s F12 medium supplemented with 10% fetal bovine serum (GIBCO), 100 U/ml penicillin and 100 μg/ml streptomycin (Invitrogen). Human embryonic kidney cells (HEK293T) and mouse embryonic fibroblasts (MEF) were cultured in Dulbecco’s modified Eagle’s medium supplemented with 10% fetal bovine serum (GIBCO), 100 U/ml penicillin and 100 μg/ml streptomycin (Invitrogen). CHO-K1 cells stably expressing CMKLR1 (Clone 53), GPR1 (Clone 8) and CCRL2 (Clone12) were cultured in presence of 10 μg/ml G418 (Invitrogen). MEF cells stably expressing chemerin receptors were cultured in presence of 10 μg/ml blasticidin (Invitrogen). The expression of each receptor was verified by saturation binding assay and/or flow cytometry. Analyses showed that the selected clones were homogeneous in terms of receptors expression and regular testing confirmed the stability of this expression over time.

### Binding assays

Binding experiments were performed as previously described [31]. Briefly, CHO-K1 cells stably expressing the receptors of interest were incubated in the assay buffer (50 mM HEPES pH 7.4, 1 mM CaCl_2_, 5 mM MgCl_2_, 250 mM sucrose, 0.5% BSA) with 0.1 nM [^125^I]-chemerin (R&DSystems, custom labelling performed by Perkin Elmer), or 0.1 nM [^125^I]-[145–157]-chemerin (Phoenix Peptides) as tracers. The tubes were incubated for 30 min at room temperature and the bound tracer was separated by filtration through GF/B filters presoaked in 1% polyethyleneimine (PEI). Filters were counted in a γ-scintillation counter. Binding parameters were determined with the PRISM software (Graphpad Softwares) using nonlinear regression applied to a single site model.

### Intracellular calcium mobilization assay

Calcium mobilization was measured by an aequorin-based assay as previously described [31]. Briefly, CHO-K1 cells stably expressing apoaequorin and the receptors of interest were incubated for 4 h in the dark in the presence of 5 μM coelenterazine *h* (Promega) and then diluted before use to reach the appropriate cell density. The cell suspension (25,000 cells/well) was added to wells containing various concentrations of chemerin and luminescence was recorded for 30 sec in an EG&G Berthold luminometer (PerkinElmer Life Sciences).

### G protein BRET assay

G protein activation was assayed by BRET as previously described [32,33]. Briefly, plasmids encoding G protein biosensors and receptors of interest were co-transfected into HEK293T cells by using the calcium phosphate method. Forty-eight hours after transfection, cells were washed twice with PBS, detached and resuspended in PBS plus 0.1% (w/v) glucose at room temperature. Cells were then distributed (80 μg of proteins per well) in a 96-well microplate (Optiplate, PerkinElmer). BRET^2^ between *R*Luc8 and GFP10 was measured 1 min after addition of coelenterazine 400a/Deep blue C (5 μM, Gentaur). BRET readings were collected using an Infinite F200 reader (Tecan). The BRET signal was calculated as the ratio of emission of GFP10 (510–540 nm) to *R*Luc8 (370–450 nm).

### β-arrestin BRET assay

β-arrestin recruitment was measured by a BRET proximity assay as previously described [28]. Briefly, plasmids encoding Rluc-β-arrestin 2 and receptors fused to Venus were cotransfected into HEK293T cells by using the calcium phosphate method. Twenty-four hours post-transfection, cells were collected and seeded in 96-well microplates (165306, Nunc) and cultured for an additional 24 h. Cells were then rinsed once with PBS and incubated in PBS plus 0.1% (w/v) glucose at 25°C to slow down kinetics of arrestin recruitment and improve temporal resolution. BRET^1^ between *R*Luc and YFP was measured after the addition of coelenterazine *h* (5 μM, Promega). Chemerin was added 5 min after coelenterazine *h* and BRET readings were collected using a Mithras LB940 Multilabel Reader (Berthold Technologies). The BRET signal was calculated as the ratio of emission of YFP (520–570 nm) to *R*Luc (370–480 nm).

### MAP kinase assay

CHO-K1 and MEF cells stably expressing receptors of interest were starved for 12–18 h in serum-free medium prior to stimulation. Cells were stimulated with 300 nM chemerin for 2 minutes, then collected by centrifugation and heated to 100°C for 5 min in 2X Laemmli sample buffer. Whole cell lysates were resolved on 10% Tris/Tricine polyacrylamide gels and transferred to nitrocellulose membranes. Phosphorylated ERK1/2 and total ERK1/2 were detected by using rabbit polyclonal anti-phospho-ERK1/2 (Cell Signaling #4370, 1:1,000) and anti-ERK1/2 (Cell Signaling #4695S, 1:2,000) antibodies. Chemiluminescent detection was performed using ECL-Plus reagent (Perkin Elmer).

### Immunofluorescence detection

*CHO-K1* cells stably expressing receptors of interest were transiently transfected with β-arrestin2-GFP using Lipofectamine 2000 (Invitrogen). Twenty-four hours post-transfection, cells were plated on 35-mm glass bottom plates. On the following day, cells were starved for at least 2 h in serum-free medium prior to stimulation. After stimulation, cells were fixed with 5% formaldehyde, and diluted in phosphate-buffered saline containing calcium and magnesium before analysis by confocal microscopy using an Axiovert 100 inverted microscope equipped with a C-Apochromat 63X/1.2 oil immersion objective (Zeiss). The 488-nm excitation beam of an Argon-Krypton laser and a 500–550-nm band-pass emission filter were used. The beam power was kept below 10% of maximal power to reduce photobleaching and phototoxicity.

### Statistical analysis

Results are expressed as arithmetic means ± SEM. Significance was determined using one-way analysis of variance, followed by Tukey's test (Prism6 software, GraphPad). For all tests, values of p<0.05 were considered as significant.

## Results

### Binding properties of CMKLR1, GPR1 and CCRL2

**Fig 1:**
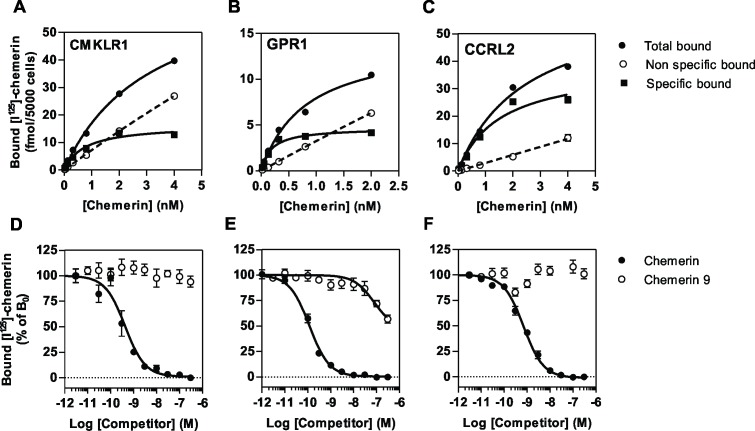
Chemerin binding assay. **A-C.** Saturation binding assay using CHO-K1 cells expressing CMKLR1 (A), GPR1 (B) or CCRL2 (C) that were incubated with increasing concentrations of [^125^I]-chemerin (total binding, ●). Non-specific binding (◯) was determined in the presence of a 100-fold excess of unlabeled chemerin and specific binding (■) was calculated as the difference. **D-F.** Competition binding assay using CHO-K1 cells expressing CMKLR1 (D), GPR1 (E) or CCRL2 (F) that were incubated with 0.1 nM [^125^I]-chemerin as tracers and increasing concentrations of unlabelled chemerin (●) or the nonapeptide chemerin 9 (◯) as competitors. The data were normalized for nonspecific binding (0%) in the presence of 300 nM chemerin, and specific binding in the absence of competitor (100%). The displayed data represent the mean ± S.E.M. of three independent experiments.

**Fig 2:**
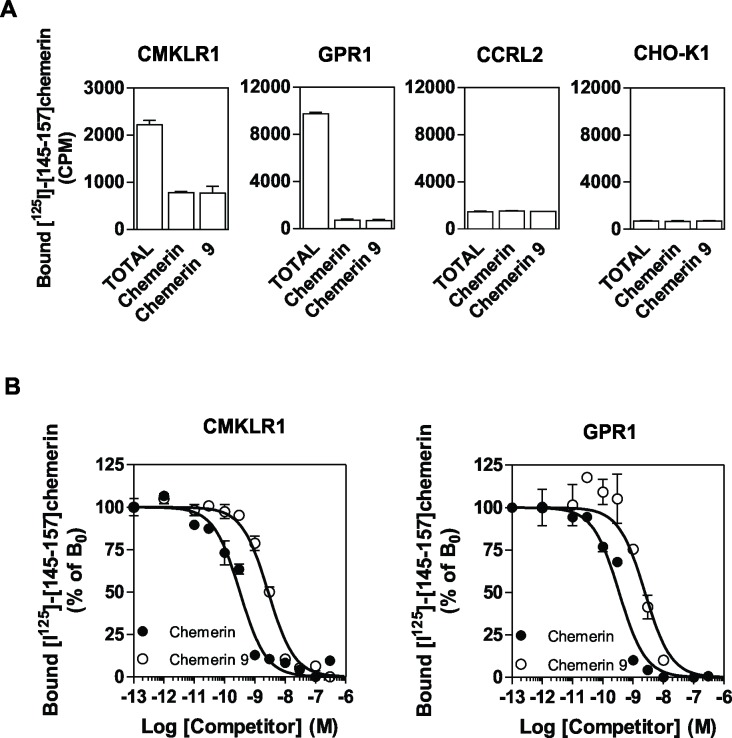
Peptide binding assay. **A** CHO-K1 cells expressing CMKLR1, GPR1 or CCRL2 were incubated with 0.1 nM [^125^I]-[145–157]-chemerin only (TOTAL) or 0.1 nM [^125^I]-[145–157]-chemerin in combination with an excess of chemerin or chemerin 9 as competitors. **B.** CHO-K1 cells expressing CMKLR1 or GPR1 were incubated with 0.1 nM [^125^I]-[145–157]-chemerin as tracer and increasing concentrations of unlabelled chemerin (●) or chemerin 9 (◯) as competitors. The data were normalized for nonspecific binding (0%) in the presence of 300 nM chemerin, and specific binding in the absence of competitor (100%). The displayed data represent the mean ± S.E.M. of three independent experiments.

**Fig 3:**
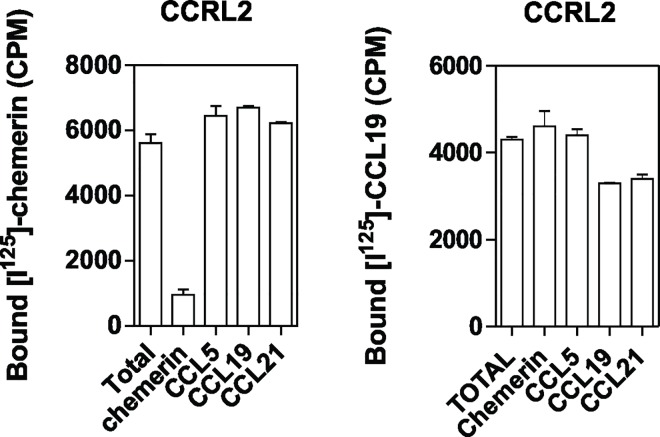
Chemokine binding to CCRL2. CHO-K1 cells expressing CCRL2 were incubated with 0.1 nM [^125^I]-chemerin (**A**) or 0.1 nM [^125^I]-CCL19 (**B**) in combination or not with an excess of unlabeled chemerin, CCL5, CCL19 or CCL21 (300nM). All points were run in triplicates (error bars indicate S.E.M.).

We first compared the binding properties of CMKLR1, GPR1 and CCRL2 by using CHO-K1 cells expressing individually each receptor. Using radio-labelled full length chemerin as tracer, we showed the selective binding of chemerin to cells expressing CMKLR1, GPR1 and CCRL2 (Fig 1). No specific binding was observed for cells expressing the related receptors GPR33, GPR44 or FPR3 used as controls or untransfected CHO-K1 cells (data not shown). In saturation binding assays, chemerin bound at single site characterized by a K_D_ of respectively 0.88 nM and 0.21 nM for CMKLR1 and GPR1, and of 2.35 nM for CCRL2 (Fig 1A–1C and Table 1). The C-terminal nonapeptide of bioactive chemerin, chemerin 9 (^149^YFPGQFAFS^157^), did not compete efficiently for the binding of full length chemerin to CMKLR1 and CCRL2. In contrast, chemerin 9 competed significantly at high concentrations (100–300 nM) for the binding of chemerin to GPR1, suggesting a stronger contribution of chemerin C-terminal moiety to GPR1 binding (Fig 1D–1F and Table 2). In order to investigate further binding properties of the chemerin 9 nonapeptide, we performed binding assays using as tracer a radio-labelled peptide derived from the C-terminus of chemerin (^125^I-[145–157]-chemerin, Phoenix Peptides). This tracer bound selectively to CMKLR1 and GPR1 but not to CCRL2 (Fig 2A and 2B). Both full size chemerin and the chemerin 9 nonapeptide competed efficiently for the binding of this tracer to CMKLR1 and GPR1 (Fig 2 and Table 2). Collectively, these results are in agreement with the current model for chemerin binding in which the core domain of chemerin binds to extracellular domains of CMKLR1 while the C-terminus of chemerin interacts with the bundle of CMKLR1 for receptor activation [1,4]. They also indicate that chemerin 9 binds to CMKLR1 and GPR1 but not to CCRL2. CCRL2 was also claimed to bind several chemokines including CCL2, CCL5, CCL7, CCL8 and CCL19 [20,22] although these data have never been confirmed by other groups and remain controversial. Here, we showed that chemokines CCL5, CCL19 and CCL21 did not competed for the binding of radio-labelled chemerin to CCRL2 (Fig 3). We also showed that the specific binding of radio-labelled CCL19 to cells expressing CCLR2 is relatively weak compared to chemerin binding and that unlabelled chemerin or CCL5 did not competed for the binding of CCL19 to CCRL2 (Fig 3). These results support rather a role of chemerin as major CCRL2 ligand.

**Table 1:** Binding parameters of CHO-K1 cells expressing human CMKLR1, GPR1 or CCRL2.

Tracer		CMKLR1	GPR1	CCRL2
[^125^I]-chemerin	**K**_**D**_ (nM)	0.88 ± 0.33	0.21 ± 0.02	2.35 ± 1.23
	**B**_**MAX**_ (fmol/5000cells)	16.4 ± 6.8	4.7 ± 0.8	38.6 ± 11.5

Binding parameters were measured on CHO-K1 cells expressing human CMKLR1, GPR1 or CCRL2. The K_D_ and B_MAX_ were obtained from curves as displayed in Fig 1. Values represent the mean ± S.E.M. of at least three independent experiments

**Table 2:** Binding parameters of CHO-K1 cells expressing human CMKLR1, GPR1 or CCRL2.

Tracer	pIC_50_	CMKLR1	GPR1	CCRL2
[^125^I]-chemerin	Chemerin	-9.4 ± 0.2	-9.9 ± 0.1	-9.1 ± 0.1
	Chemerin 9	ND	< 7	ND
[^125^I]-[145–157]-chemerin	Chemerin	-9.5 ± 0.1	-9.5 ± 0.1	ND
	Chemerin 9	-8.6 ± 0.1	-8.5 ± 0.1	ND

Binding parameters were measured on CHO-K1 cells expressing human CMKLR1, GPR1 or CCRL2. The pIC_50_ were obtained from competition binding experiments as displayed in Figs 1 and 2. Values represent the mean ± S.E.M. of at least three independent experiments. ND, not detected.

### Activation of G protein subtypes by CMKLR1

**Fig 4:**
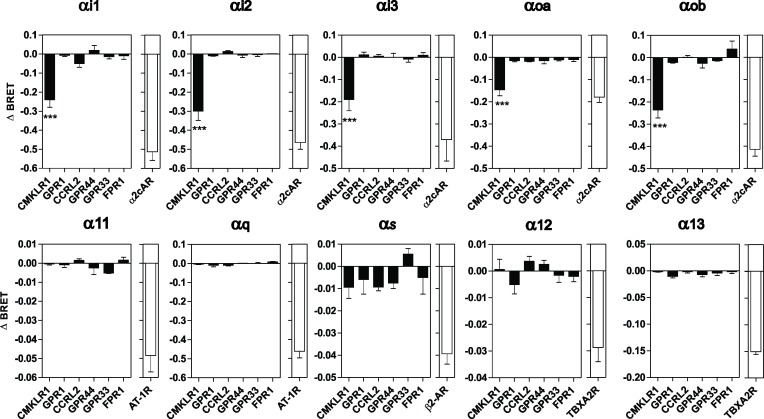
Determination of the range of G proteins activated by chemerin receptors. Real-time measurement of BRET signal in HEK293T cells coexpressing G protein biosensors and CMKLR1, GPR1, CCRL2, GPR44, GPR33 or FPR1, following stimulation for 1 minute by 100 nM chemerin. Results are expressed as the difference in BRET signals measured in the presence and absence of chemerin. As controls, cells expressing the α2c adrenergic receptor (G_αi/o_) were stimulated with UK14304; cells expressing the AT1 angiotensin receptor (G_α11/q_) were stimulated with angiotensin II; cells expressing the β2 adrenergic receptor (G_αs_) were stimulated with isoproterenol; cells expressing thromboxane A2 receptor (G_α12/13_) were stimulated with U46619. Data represent the mean ± S.E.M. of three to six independent experiments. Statistical significance was assessed using Tukey's test (***P < 0.0001).

**Fig 5:**
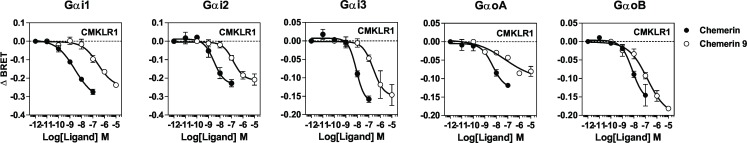
Activation of Gαi and Gαo by CMKLR1. Real-time measurement of BRET signal in HEK293T cells expressing G protein biosensors and CMKLR1, following stimulation for 1 minute with increasing concentrations of chemerin (●) or the chemerin-9 nonapeptide (◯). Results are expressed as the difference in BRET signals measured in the presence and absence of chemerin. Data represent the mean ± S.E.M. of three independent experiments.

We next investigated the panel of G proteins activated upon binding of chemerin to CMKLR1, GPR1 and CCRL2 by using BRET-based biosensors monitoring conformational changes of G proteins. This technology relies on the interdomain movement that occurs within heterotrimeric G proteins upon GDP/GTP exchange, resulting in a decrease of the BRET signal between probes inserted within Gα and Gγ subunits. This technology detects therefore an early signaling event that occurs shortly after receptor stimulation, and allows discriminating the G protein subtypes activated by a receptor [28,32,33]. Chemerin binding to CMKLR1 triggered a significant activation of the three Gα_i_ subtypes (Gα_i1_, Gα_i2_, Gα_i3_) and the two Gα_o_ isoforms (Gα_oa_, Gα_ob_) (Fig 4). The BRET signals were similar to those detected upon stimulation of the α_2C_ adrenergic receptor used as reference for Gα_i/o_ activation. Cells expressing the Gαi/o biosensors with GPR44/CRTH2, GPR33 or FPR1 were used as negative controls, and generated signals of much weaker magnitude. Of note, no significant BRET signal was detected for the Gα_q_, Gα_11_, Gα_s,_ Gα_12_ or Gα_13_ proteins (Fig 4). By comparison, stimulation of receptors known to couple efficiently to Gα_q/11_ (AT1-R), Gα_s_ (β2A-R) or Gα_12/13_ (TPα-R) generated signals of much higher amplitude. In contrast to CMKLR1, none of the G proteins tested were activated by GPR1 or CCRL2 upon chemerin stimulation, the weak BRET signals detected being similar to those of the negative controls, confirming that GPR1 and CCRL2 do not efficiently activate G proteins. Chemerin binding to CMKLR1 activated Gα_i/o_ with calculated pEC_50_ values compatible with the affinity of chemerin for CMKLR1 (Fig 5 and Table 3). In contrast, binding of chemerin 9 activated Gα_i/o_ with a reduced potency compared to chemerin (Fig 5 and Table 3). Collectively, these data indicate that chemerin induces the activation of Gα_i/o_ proteins merely through CMKLR1 binding.

**Table 3:** Signaling parameters of CMKLR1 and GPR1.

Signaling parameters of CMKLR1 and GPR1														
	Gαi1		Gαi2		Gαi3		Gαoa		Gαob		β-Arr1		β-Arr2	
CMKLR1	pEC_50_	E_MAX_	pEC_50_	E_MAX_	pEC_50_	E_MAX_	pEC_50_	E_MAX_	pEC_50_	E_MAX_	pEC_50_	E_MAX_	pEC_50_	E_MAX_
**Chemerin**	-8.55 ± 0.21	0.35 ± 0.07	-8.55 ± 0.30	0.26 ± 0.05	-8.09 ± 0.14	0.18 ± 0.02	-8.28 ± 0.21	0.12 ± 0.02	-8.12 ± 0.31	0.17 ± 0.04	-7.49 ± 0.11	0.52 ± 0.04	-8.00 ± 0.09	0.38 ± 0.02
**Chemerin 9**	-6.85 ± 0.19	0.21 ± 0.02	-6.85 ± 0.19	0.21 ± 0.01	-6.62 ± 0.27	0.15 ± 0.02	-6.92 ± 0.27	0.09 ± 0.02	-6.97 ± 0.15	0.18 ± 0.02	ND	ND	ND	ND
**GPR1**														
**Chemerin**	ND	ND	ND	ND	ND	ND	ND	ND	ND	ND	-8.01 ± 0.10	0.10 ± 0.01	-7.87 ± 0.11	0.20 ± 0.01
**Chemerin 9**	ND	ND	ND	ND	ND	ND	ND	ND	ND	ND	-6.49 ± 0.10	0.11 ± 0.01	-7.47 ± 0.11	0.14 ± 0.01

**Fig 6:**
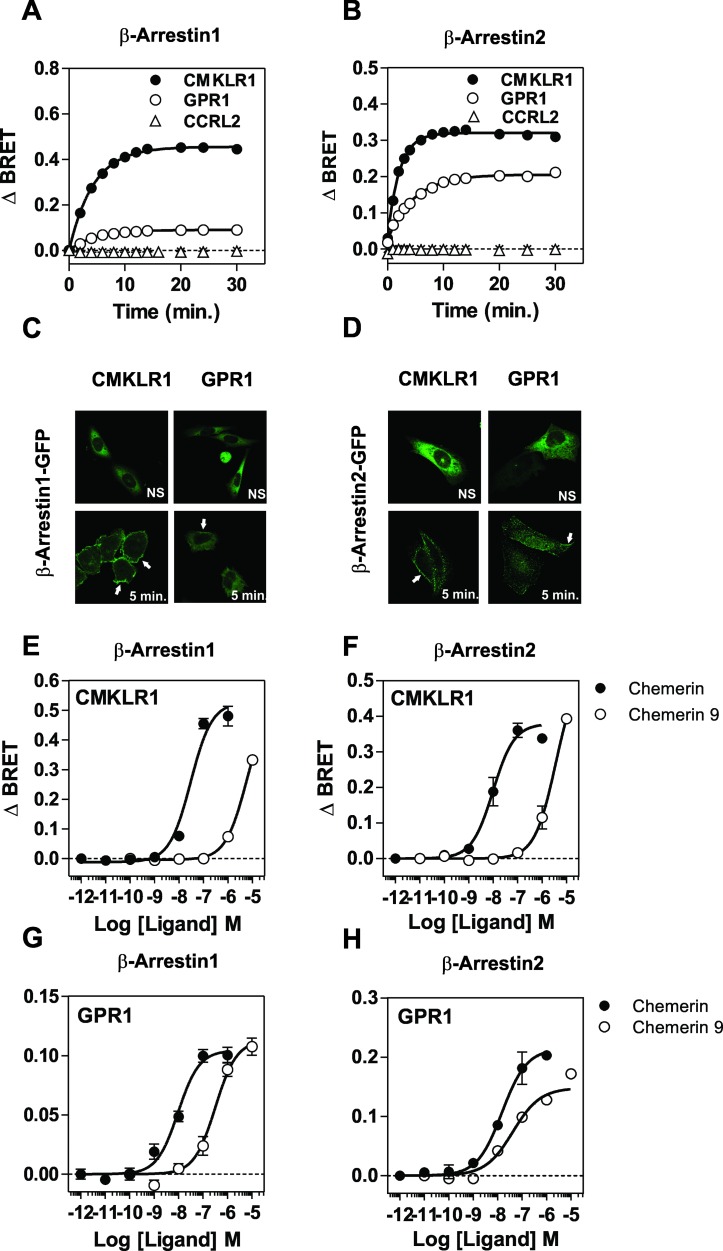
Recruitment of β-arrestins by CMKLR1 and GPR1. **A.** Real-time measurement of BRET signal in HEK293T cells expressing either β-arrestin1-*R*Luc only (✳) or together with CMKLR1-Venus (●), GPR1-Venus (◯) or CCRL2-Venus (△), following stimulation by 100 nM chemerin. **B.** Real-time measurement of BRET signal in HEK293T cells expressing either β-arrestin2-*R*Luc only (✳) or together with CMKLR1-Venus (●), GPR1-Venus (◯) or CCRL2-Venus (△), following stimulation by 100 nM chemerin. **C-D** Localization of β-arrestin in cells coexpressing β-arrestin1-EYFP (C) or β-arrestin2-GFP and CMKLR1 or GPR1, before (NS) and 5 minutes after stimulation with 100 nM chemerin. **E-F.** Real-time measurement of BRET signal in HEK293T cells expressing β-arrestin1-*R*luc and CMKLR1-Venus or GPR1-Venus following stimulation with increasing concentrations of chemerin (●) or the chemerin 9 nonapeptide (◯). **G-H** Real-time measurement of BRET signal in HEK293T cells expressing β-arrestin2-*R*luc and CMKLR1-Venus or GPR1-Venus following stimulation with increasing concentrations of chemerin (●) or the chemerin 9 nonapeptide. Results of BRET experiments are expressed as the difference in BRET signals measured in the presence and absence of chemerin. Data represent the mean ± S.E.M. of three independent experiments.

Signaling parameters were measured on HEK293T cells expressing CMKLR1 or GPR1. The pEC_50_ and E_MAX_ values were obtained from experiments displayed in Figs 5 and 6. E_MAX_ values corresponds to BRET_MAX_ values. Values represent the mean ± S.E.M. of at least three independent experiments. ND, not determined

### β-arrestins recruitment by CMKLR1 and GPR1

**Fig 7:**
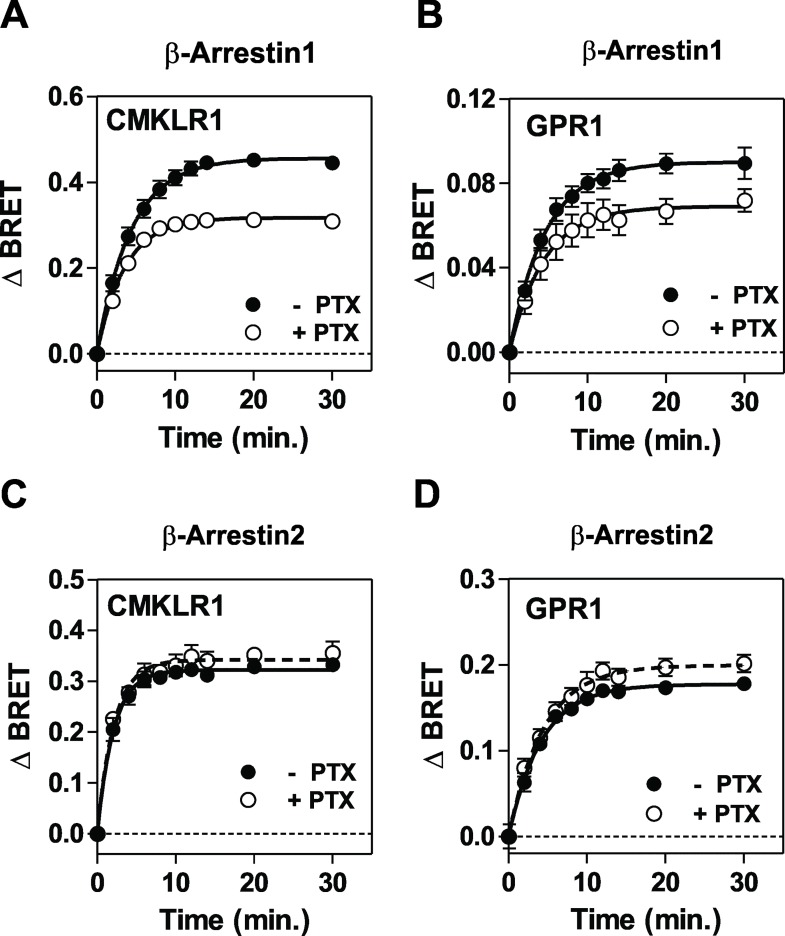
Impact of *Pertussis toxin* on arrestins recruitment to CMKLR1 and GPR1. Real-time measurement of BRET signal in HEK293T cells expressing β-arrestin1-*R*luc (**A**-**B**) or β-arrestin2-*R*luc (**C**-**D**) in combination with CMKLR1-Venus or GPR1-Venus, following stimulation with 100 nM chemerin in the absence (●) or the presence of Pertussis toxin (PTX, ◯). Results of BRET experiments are expressed as the difference in BRET signals measured in the presence and absence of chemerin. Data represent the mean ± S.E.M. of three independent experiments.

Although β-arrestins were initially considered as molecules that terminate GPCR signaling by uncoupling receptors from G proteins and by promoting internalization, they were also shown to activate pathways on their own by serving as a "scaffold" for signaling proteins [34]. With the aim of comparing β-arrestins recruitment by chemerin receptors, we first relied on a BRET proximity assay measuring the energy transfer between β-arrestins-Rluc and chemerin receptors fused to the yellow fluorescent protein Venus. Due to the rapid recruitment of arrestins at 37°C, assays were performed at 25°C to slower kinetics and gain in temporal resolution. Binding of chemerin induced a progressive increase in energy transfer between β-arrestin1-Rluc or β-arrestin2-Rluc and CMKLR1-Venus, indicating efficient recruitment of β-arrestins to CMKLR1 (Fig 6A and 6B). Chemerin also induced an increase in energy transfer between β-arrestin1-Rluc or β-arrestin2-Rluc and GPR1-Venus, although to various degree (Fig 6 A and 6B). In contrast, energy transfer between β-arrestins-Rluc and CCRL2-Venus remained constant whether cells were stimulated or not, indicating that CCRL2 does not recruit β-arrestins (Fig 6A and 6B). Fluorescent microscopy confirmed that chemerin induces the redistribution of β-arrestin1-EYFP and β-arrestin2-GFP from the cytosol to plasma membrane (arrows) and punctuated fluorescent structures in cells expressing CMKLR1 or GPR1, thought the redistribution is barely detectable in for β-arrestin1-EYFP in cells expressing GPR1 (Fig 6C and 6D). The BRET assay also showed that CMKLR1 recruits β-arrestins with a faster kinetics compared to GPR1. The maximal BRET values (BRET_MAX_) reached upon CMKLR1 stimulation were also higher than the BRET_MAX_ measured upon GPR1 stimulation. This difference might reflect a higher efficiency of arrestins recruitment by CMKLR1 or distinct conformations of receptor-arrestin complexes. Nevertheless, the recruitment of β-arrestin1 and 2 by CMKLR1 and GPR1 occurred with similar potencies (Fig 6E–6H, Table 3). Interestingly, binding of the nonapeptide chemerin 9 to CMKLR1 induced β-arrestins activation with 100-fold lower potency than full size chemerin, whereas chemerin and chemerin 9 induced the recruitment of β-arrestins with much closer potencies through GPR1 (Fig 6 E–6H, Table 3). Finally, we showed that pretreatment of cells with *Pertussis* toxin (PTX), which causes ADP-ribosylation of Gα_i/o_ proteins, inhibits partially β-arrestin1 recruitment to CMKLR1 and GPR1. This result may appear puzzling for GPR1, but we cannot exclude formally that a weak activation of G proteins contributes to GPR1 signaling. In contrast, PTX did not inhibit β-arrestin2 recruitment to CMKLR1 and GPR1, suggesting that β-arrestin2 recruitment occurs independently of Gα_i/o_ activation (Fig 7).

### Down-regulation of CMKLR1 and GPR1

**Fig 8:**
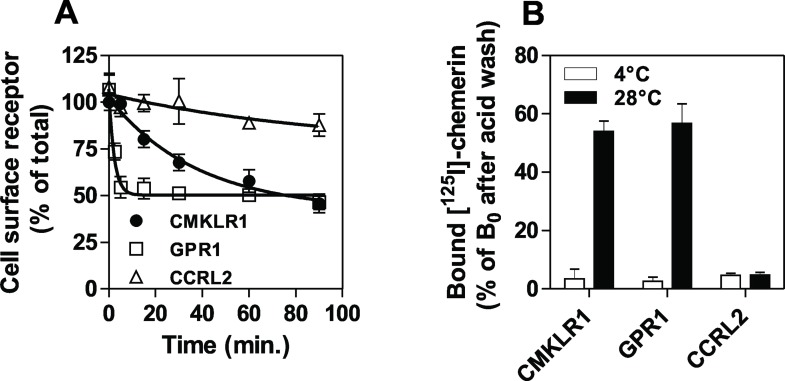
**Down-regulation of CMKLR1 and GPR1 A.** CHO-K1 cells expressing chemerin receptors were incubated with 100 nM chemerin for various periods of time. Cell surface receptor was detected by flow cytometry using a saturating concentration of antibodies specific for CMKLR1 (●), GPR1 (◯) or CCRL2 (△). Results were normalized for the fluorescence of unstimulated cells (100%) and for background fluorescence (0%). Data represent the mean ± S.E.M. of three independent experiments. **B.** CHO-K1 cells expressing chemerin receptors were first incubated with ^125^I-chemerin at 4°C and washed with binding buffer containing 500 mM NaCl to eliminate the unbound tracer. Then, cells were either left at 4°C or shifted to 28°C to allow receptor internalization. After 90 minutes, cells were acid-washed and the amount of radioactivity remaining associated with the cells was measured. Data represent the mean ± S.E.M. of three independent experiments.

The ability of GPR1 to recruit arrestin and to activate weakly G proteins suggests that it may act as a decoy receptor. With the aim of comparing the capacity of chemerin to induce receptor endocytosis, we first measured the chemerin-induced down-regulation of the receptors by FACS. Upon chemerin stimulation, CMKLR1 disappeared progressively from the cell surface while GPR1 was rapidly down-regulated (t_1/2_ = 26 min and 1.6 min. respectively, Fig 8). In contrast, chemerin binding to CCRL2 induced a very weak down-regulation of the receptor (t_1/2_ > 1h, Fig 8A). Next, we compared the fate of bound chemerin by measuring the amount of chemerin internalized by the cells. Radiolabelled chemerin was incubated for 30 minutes with cells expressing chemerin receptors at 4°C, a temperature that slows down internalization. Thereafter, cells were either kept at 4°C or incubated at 28°C to allow receptor internalization, and after 45 minutes, cells were subjected to an acid-wash to eliminate chemerin bound to the cell surface. When cells were kept at 4°C, most of the cell-associated radioactivity was lost, indicating that chemerin was efficiently stripped out of the cell surface by the acid-wash (Fig 8B). In contrast, upon incubation at 28°C, about half of the radioactivity remained associated to cells expressing CMKLR1 or GPR1, indicating that CMKLR1 and GPR1 internalized bound chemerin. By comparison, nearly all the radioactivity associated to cells expressing CCLR2 was lost after the acid-wash procedure, confirming that the chemerin-CCRL2 complex remains at the cell surface.

### Signaling properties of CMKLR1 and GPR1

**Fig 9:**
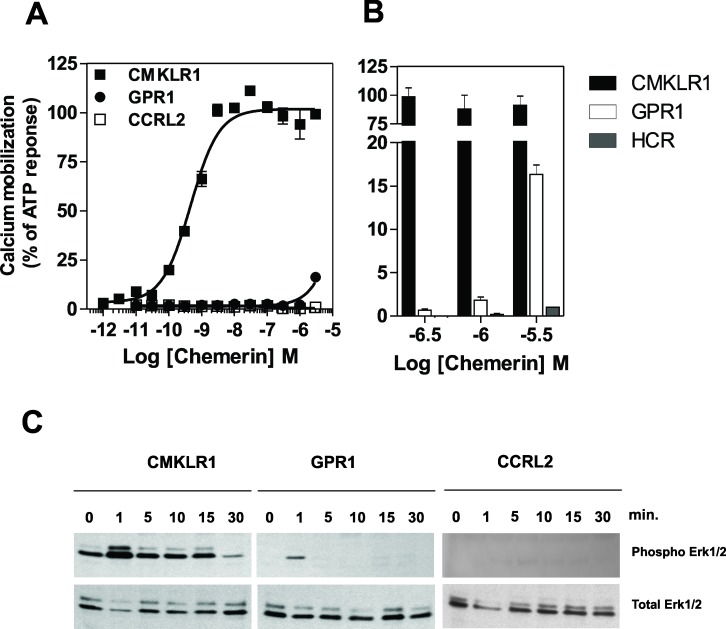
Functional response of chemerin receptors. **A.** Calcium mobilization was measured in CHO-K1 cells using the aequorin-based functional assay. Cells expressing chemerin receptors were stimulated with increasing concentrations of chemerin and luminescence was recorded for 30 s. The results were normalized for the basal luminescence of the cells in absence of agonist (0%) and the maximal response obtained for each cell line with 10 μM ATP (100%). **B.** Enlarged panel derived from Fig 9 A showing that GPR1 signal triggered by 3 μM chemerin accounts for about 15% of the CMKLR1 signal. Data represent the mean ± S.E.M. of three independent experiments **C.** Immunoblot detection of phosphorylated ERK1/2 MAP kinases revealed with anti-phospho ERK1/2 (upper panel). CHO-K1 cells expressing chemerin receptors were stimulated with 300 nM chemerin for various times. Detection of total ERK1/2 by Western blotting was used to ascertain that an equal amount of material was loaded in each lane (lower panel). A typical experiment out of three performed independently is shown.

**Fig 10:**
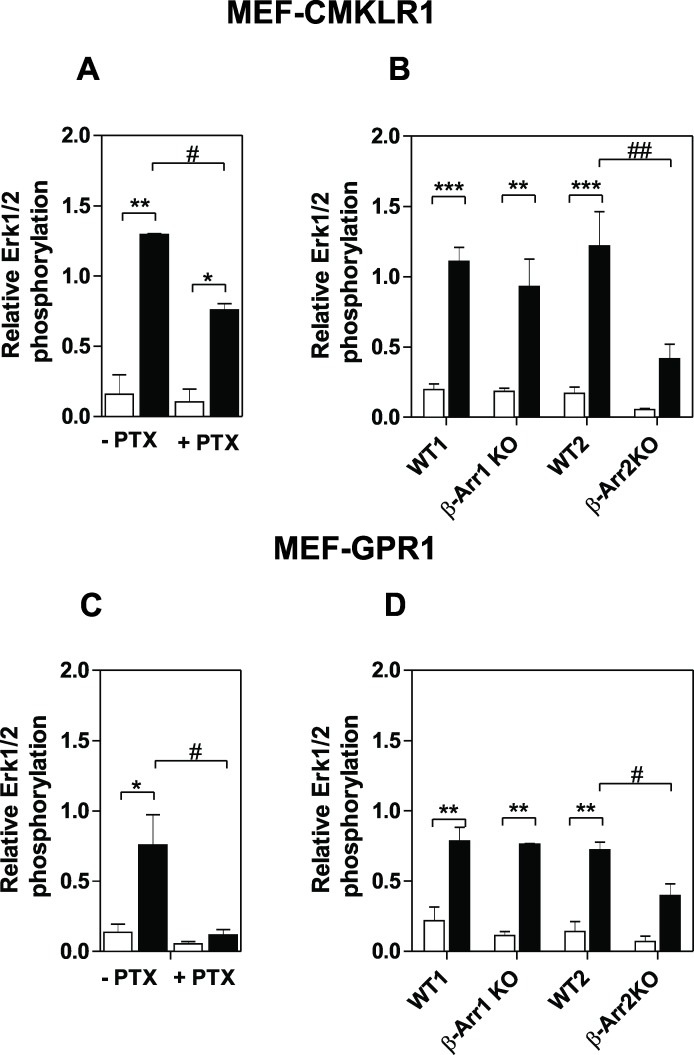
Contribution of Gi/o proteins and arrestins to CMKLR1- and GPR1-mediated ERK1/2 phosphorylation. Mouse embryonic fibroblasts (MEF) expressing CMKLR1 or GPR1 were stimulated with 300 nM chemerin (wlack bars) or buffer only (white bars) for two minutes and phosphorylation of ERK1/2 estimated by Western blotting. Results are expressed as the ratio between the amounts of phospho-ERK1/2 and total ERK1/2 following quantification. Data represent the mean ± S.E.M. of three independent experiments. MEF cells were derived form β-Arr1 KO and WT1 or β-Arr2 KO and WT2 siblings. Data represent the mean ± S.E.M. of three independent experiments. Statistical significance was assessed using Tukey's test (***P < 0.0001; ** and ^##^ *P<* 0.001; * and ^#^ *P<* 0.01).

The recruitment of arrestin by CMKLR1 and GPR1 also suggests that it may contribute to signaling through G protein-independent pathways. Therefore, we re-investigated the signaling properties of these two receptors and, in perfect agreement with previous studies, showed that chemerin binding to CMKLR1 induced a strong calcium mobilization as well as the phosphorylation of MAP kinases ERK1/2 (Fig 9). In contrast, chemerin binding to GPR1 induced a barely detectable calcium mobilization that peaks only at about 15% of the CMKLR1 response for 3 μM chemerin (Fig 9A). Chemerin binding to GPR1 also induced ERK1/2 phosphorylation, although to a much weaker extent than CMKLR1 (Fig 9B). These results argue for GPR1 being a receptor of high affinity for chemerin but with a weak signaling capacity compared to CMKLR1. By comparison, binding of chemerin to CCRL2 generated no detectable signals (Fig 9). We next investigated the contribution of Gαi/o proteins and arrestins to ERK1/2 phosphorylation by using mouse embryonic fibroblast cells (MEF) deficient for the expression of arrestins (β-Arr1^-/-^ and β-Arr2^-/-^) and stably expressing CMKLR1 or GPR1. Binding of chemerin to CMKLR1 and GPR1 induced the phosphorylation of ERK1/2 in wild-type MEF cells in perfect agreement to what we reported in CHO-K1 cells. Phosphorylation of ERK1/2 was partially inhibited by pre-treatment of cells with *Pertussis* toxin (PTX), indicating that Gαi/o proteins contribute to ERK1/2 activation (Fig 10A and 10C). This result may appear somehow paradoxical for GPR1. However, we cannot rule out that the weak activation of G proteins by GPR1 contributes to ERK1/2 phosphorylation. We also must take into consideration that these assays display different levels of amplification and were performed in different cell types. We also showed that ERK1/2 activation is reduced in β-Arr2^-/-^ cells compared to β-Arr1^-/-^ or control cells, demonstrating that CMKLR1 and GPR1 also activate ERK1/2 phosphorylation in a β-arrestin2-dependent manner (Fig 10B and 10D). Collectively, these results show that the recruitment of β-arrestin2 contributes to the activation of ERK1/2 and that CMKLR1 and GPR1 promote ERK1/2 activation most probably through non-exclusive G protein and β-arrestin-dependent pathways.

## Discussion

**Fig 11:**
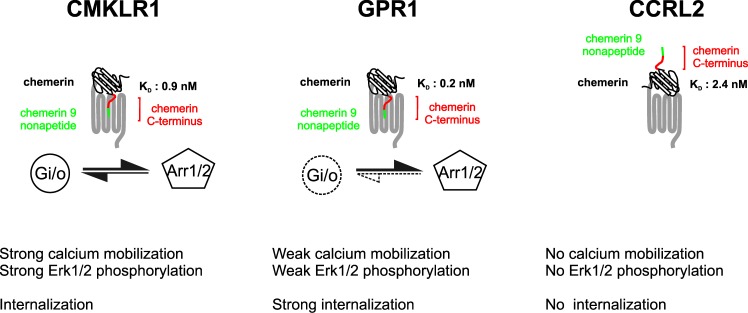
Overview of the three chemerin receptors. Binding of chemerin to CMKLR1 leads to the activation of Gi/o proteins and arrestins as well as to calcium mobilization, Erk1/2 phosphorylation and internalization of the chemerin–receptor complex. Binding of chemerin to GPR1 leads mainly to the activation arrestins, although we cannot exclude contribution of G protein to GPR1 signaling. A weak calcium mobilization and Erk1/2 phosphorylation is also detected in response chemerin. GPR1 also internalizes efficiently in response to chemerin. In contrast, CCRL2 binds efficiently chemerin but does not signal nor internalize. The current hypotheses is that CCRL2 might present chemerin C-terminus (in red) to nearby cells expressing functional receptors or play the role of chemerin scavenger.

Chemerin is a small chemoattractant protein found in the circulation and in inflammatory fluids that mediates its effects through CMKLR1. Besides CMKLR1, two other receptors, GPR1 and CCRL2, have been shown to bind chemerin, but the pharmacology and signaling properties of these chemerin receptors has been much less characterized. In this study, we compared the binding properties of chemerin receptors and showed that chemerin binds to all three receptors with high affinity, although GPR1 binds chemerin with the highest affinity (K_D_: 0.21 nM) and CCRL2 with the lowest (K_D_: 2.35 nM) (Fig 11). We also showed that the contribution of chemerin C-terminus to the interaction varies for each receptor. The radiolabelled peptide ^145^PHSFYFPGQFAFS^157^, derived from chemerin C-terminus, binds well to CMKLR1 and GPR1 but not to CCRL2. When chemerin binds to CCRL2, the C-terminal peptide might therefore remain accessible for interaction with CMKLR1-expressing neighbour cells, as hypothesized in a previous study [11] (Fig 11). However, we can’t rule out that the atypical receptor CCRL2 may also serve as a scavenger for chemerin. Using this radiolabelled peptide, we also showed that the chemerin 9 nonapeptide (^149^YFPGQFAFS^157^) binds with a same apparent affinity to CMKLR1 and GPR1. In contrast, chemerin 9 did not compete for the binding of full length chemerin to CMKLR1 while it competed at high concentrations for the binding chemerin to GPR1. Binding of chemerin to GPR1 may thus involve a higher contribution of the C-terminal moiety than for CMKLR1 binding. Whether this different mode on interaction affects the activation of G proteins by GPR1 is not known. A likely explanation for the reduced G protein activation by GPR1 is the presence of an altered D(R/H)Y motif at the end of TM3 [35]. We also compared the functional properties of chemerin receptors by using BRET-based biosensors directly monitoring the activation of G proteins. Binding of chemerin to GPR1 or CCRL2 did not induce activation of any of the G proteins tested. In contrast, binding of chemerin to CMKLR1 induced the activation of the three Gαi subtypes and the two Gαo isoforms with similar potencies (Fig 11). The panel of G proteins activated by CMKLR1 is similar to that of the chemokine receptors CXCR4 or CCR7, but restricted compared to CCR2 or CCR5 that also activate the Gα12 protein [28]. In addition, we showed that the chemerin 9 peptide triggers the activation of the same panel of G proteins although with a reduced potency. These results are consistent with the difference observed previously between full size chemerin and the chemerin 9 nonapeptide in a calcium mobilization assay [4]. However, the potency of both agonists was lower in the G protein assays than in the calcium-mobilization assay that involves different levels of amplification along the pathway. By using BRET-proximity assays, we also showed that chemerin induces the recruitment of β-arrestins to CMKLR1 and GPR1, though to various degree (Fig 11). Therefore, compared to CMKLR1, GPR1 recruits β-arrestins but activates weakly G proteins. This behaviour is in line with the atypical structure of GPR1 which does not display the canonical DRY motif required for G protein coupling. However, the partial inhibition of β-arrestin1 and Erk1/2 activation by PTX suggests that G protein contribute to GPR1 signaling. The chemerin 9 peptide also induces the recruitment of β-arrestins to CMKLR1 but with a 100-fold reduced potency compared to chemerin. In contrast, binding of chemerin and chemerin 9 peptide to GPR1 triggered the activation of β-arrestins with much closer potencies. The chemerin 9 exhibits thus a higher propensity to activate arrestins when bound to GPR1. Whether these results reflect a true signaling bias should however be considered with caution as we also showed that the C-terminal peptide contributes more significantly to the binding of chemerin to GPR1. We also showed in this study that chemerin induced the down-regulation of both CMKLR1 and GPR1 (Fig 8). However, the kinetics of down-regulation and arrestins recruitment did not correlate, suggesting that other mechanisms likely contribute to receptor down-regulation. The down-regulation kinetics is also much faster for GPR1, which supports a putative role of GPR1 as a decoy receptor. In line with this hypothesis, we confirmed that radiolabelled chemerin is efficiently internalized by cells expressing CMKLR1 or GPR1. It has also been anticipated that arrestin interaction with CMKLR1 and GPR1 might contribute to G protein-independent signaling. We showed that binding of chemerin to CMKLR1 and GPR1 triggers the phosphorylation of ERK1/2, although to a different extent, and that this activation requires β-arrestin2 but not β-arrestin1. These results constitute the first indication that GPR1 may activate downstream signaling cascades through the recruitment of a selective arrestin. It should be noted that the activation of ERK1/2 by GPR1 is relatively weak and that other downstream signaling events might be activated by GPR1. Collectively, these data suggest that phosphorylation of ERK1/2 by CMKLR1 and GPR1 involves non-exclusively G protein and β-arrestin2-dependent signaling pathways. Whether some signaling molecules are activated selectively by β-arrestin2 remains thus an open question that will require further analysis.

In summary, we show that chemerin elicits distinct responses according to the bound receptor. Stimulation of CMKLR1 triggers G proteins activation, β-arrestins recruitment and receptor internalization, whereas stimulation of GPR1 is strongly biased towards β-arrestins recruitment and receptor internalization. In contrast, stimulation of CCRL2 does not seem to promote any signaling in the cells, and does not induce receptor internalization. We also show that β-arrestin2 recruitment by CMKLR1 and GPR1 participates in Erk1/2 activation, supporting the existence of a β-arrestin2-dependent signaling pathway activated by chemerin receptors. These results constitute the first indication of the existence of a β-arrestin2-dependent signaling at chemerin receptors. Whether this atypical signaling also exists in cells which endogenously express CMKLR1 or GPR1 remains to be determined precisely. Further experiments will also be needed to address remaining questions regarding chemerin receptors such as the importance of chemerin processing for GPR1 and CCRL2 binding, the existence of an atypical signaling downstream CCRL2 or the consequence of receptor homo- and hetero-oligomerization as oligomerization may impact on the signaling of chemerin receptors as reported for other GPCRs [36].
